# Supplementary material for: Prediction of cognitive impairment through speech data analysis: A comparative evaluation of deep learning models
Source: PLoS One. 2026 May 21;21(5):e0349412. doi: 10.1371/journal.pone.0349412 (PMC13193450; doi:10.1371/journal.pone.0349412)
Supplement: Attachment — Submitted filename: Revised_decision.pdf [file pone.0349412.s003.pdf]

The authors have made substantial revisions to the manuscript and conducted additional analysis that address my concerns. The manuscript is now appropriate for publication, though limitations remain.

Recommendation:
Despite these limitations, I recommend the manuscript for publication.
