# Supplementary material for: Prediction of cognitive impairment through speech data analysis: A comparative evaluation of deep learning models
Source: PLoS One. 2026 May 21;21(5):e0349412. doi: 10.1371/journal.pone.0349412 (PMC13193450; doi:10.1371/journal.pone.0349412)
Supplement: Attachment — Submitted filename: Review_PONE-D-25-44894.docx [file pone.0349412.s001.docx]

This study addresses the critical need for no-invasive diagnostic tools for cognitive decline, by evaluating multiple deep learning architectures applied to dataset from “Cognitive Impairment Diagnosis Voice/Conversation”. Using this dataset the study compares the efficacy of 1D CNN, AST and Wav2Vec2.0 in classifying binary tasks: normal cognition (NC) vs. mild cognitive impairment (MCI) and NC vs. Alzheimer’s disease (AD). The paper concludes that Wav2Vec2.0 outperforms other architectures, particularly in the NC vs. AD classification task.

The primary strength of this work lies in systematic, side by side evaluation of disparate modeling approaches (eg. VGG-19, ResNet, transformer-based models) to audio models providing a clear hierarchy of effectiveness. In particular, the results reveal that self-supervised speech representation model such as Wav2Vec2.0 is better suited for cognitive changes in speech.

I had a few comments that I believe would improve the paper and its applicability.

1. This study prioritizes female voices due to lower volume of male recordings. Although this choice is understandable, it raises concern about generalizability of the results. It would have been interesting to see how sex-specific vocal characteristics might influence model performance and whether conclusions are expected to hold for equally mixed/male-dominated cohorts.
2. If I could make out correctly, the results aggregate the performance across the 11 different tasks (eg. Sentence repetition, language fluency). In the limiting case what would be the effect of analyzing these individually? Some tasks (eg. storytelling) are biologically more relevant for cognitive decline than other (Image description). I was wondering which specific tasks account for higher Wav2Vec2.0. This in turn could streamline future data collections.
3. Finally, the model performance is reported using across five-fold cross-validation. Given relatively modest performance differences between some models, reporting statistical significance testing/confidence interval would help clarify the robustness.

Overall, this manuscript presents a well-executed study using speech dataset and deep learning techniques. The comparative analysis is valuable contribution to the field of digital biomarker. I believe addressing above points would strengthen the paper.
