# Supplementary material for: Prediction of cognitive impairment through speech data analysis: A comparative evaluation of deep learning models
Source: PLoS One. 2026 May 21;21(5):e0349412. doi: 10.1371/journal.pone.0349412 (PMC13193450; doi:10.1371/journal.pone.0349412)
Supplement: Attachment — Submitted filename: Response to Reviewers_v2.docx [file pone.0349412.s004.docx]

**Journal Requirements:**

1. When submitting your revision, we need you to address these additional requirements.

Please ensure that your manuscript meets PLOS ONE's style requirements, including those for file naming. The PLOS ONE style templates can be found at

<https://journals.plos.org/plosone/s/file?id=wjVg/PLOSOne_formatting_sample_main_body.pdf> and

<https://journals.plos.org/plosone/s/file?id=ba62/PLOSOne_formatting_sample_title_authors_affiliations.pdf>

**Response**:  Thank you for your valuable feedback on our manuscript. We have carefully revised the manuscript according to the reviewers' comments. The specific changes are as follows:

Manuscript Title: The title has been updated to Sentence case as requested.

Author Information: Academic degrees (MD, PhD) have been removed, and the corresponding author's information has been simplified to include only the email address and initials.

Section Headings: We have adjusted the font sizes (18pt and 16pt) and applied Sentence case formatting to all section titles.

Figure Captions: All figure captions have been repositioned to appear immediately following their first citation within the text.

We hope these revisions meet the journal's requirements.

2. Please note that PLOS One has specific guidelines on code sharing for submissions in which author-generated code underpins the findings in the manuscript. In these cases, we expect all author-generated code to be made available without restrictions upon publication of the work. Please review our guidelines at <https://journals.plos.org/plosone/s/materials-and-software-sharing#loc-sharing-code> and ensure that your code is shared in a way that follows best practice and facilitates reproducibility and reuse.

**Response:** While we fully support PLOS ONE's software sharing guidelines, our author-generated code (utilized for audio preprocessing and deep learning model training) was developed in collaboration with a commercial partner. Due to the inclusion of proprietary company know-how and intellectual property restrictions, we are unable to make the source code publicly available in an unrestricted repository. However, to facilitate the rigorous review process and ensure methodological transparency, we are fully prepared to provide the code confidentially to the reviewers.

Private link: <https://figshare.com/s/3d395292a035369d4bac>

3. Please include your tables as part of your main manuscript and remove the individual files. Please note that supplementary tables (should remain/ be uploaded) as separate "supporting information" files.

**Response:** All tables have been directly inserted into the main manuscript text rather than uploaded as separate individual files.

4. We note that the grant information you provided in the ‘Funding Information’ and ‘Financial Disclosure’ sections do not match.

When you resubmit, please ensure that you provide the correct grant numbers for the awards you received for your study in the ‘Funding Information’ section.

**Response:** We sincerely apologize for the initial discrepancy. As requested, we have carefully amended the Funding Statement within our Cover Letter to explicitly list all precise sources of support. The updated statement now explicitly includes the Tech Incubator Program for Startup Korea (TIPS), the Korea Technology and Information Promotion Agency for SMEs (TIPA), and the Ministry of SMEs and Startups (MSS).

“This work was supported by the Tech Incubator Program for Startup Korea (TIPS) through the Korea Technology and Information Promotion Agency for SMEs (TIPA) funded by the Ministry of SMEs and Startups (MSS, Republic of Korea) (Project No. S3079103) and the Cooperative Research Program for Agriculture Science and Technology Development Rural Development Administration (Project No. PJ01712403). There was no additional external funding received for this study.”

5. Thank you for stating in your Funding Statement:

G.Y. was supported by grants from the Ministry of SMEs and Startups (Project Number: S3079103). Y.C.Y acknowledges funding from the Cooperative Research Program for Agriculture Science and Technology Development Rural Development Administration (Project No. PJ01712403).

No, the sponsors/funders had no role in the study design, data collection and analysis, decision to publish, or preparation of the manuscript. The research was conducted independently by the authors.

Please provide an amended statement that declares *all* the funding or sources of support (whether external or internal to your organization) received during this study, as detailed online in our guide for authors at <http://journals.plos.org/plosone/s/submit-now>.  Please also include the statement “There was no additional external funding received for this study.” in your updated Funding Statement.

Please include your amended Funding Statement within your cover letter. We will change the online submission form on your behalf.

**Response:** We have updated the funding statement in the manuscript and cover letter.

**“Funding**

This work was supported by the Tech Incubator Program for Startup Korea (TIPS) through the Korea Technology and Information Promotion Agency for SMEs (TIPA) funded by the Ministry of SMEs and Startups (MSS, Republic of Korea) (Project No. S3079103) and the Cooperative Research Program for Agriculture Science and Technology Development Rural Development Administration (Project No. PJ01712403). There was no additional external funding received for this study.”

6. Thank you for uploading your study's underlying data set. Unfortunately, the repository you have noted in your Data Availability statement does not qualify as an acceptable data repository according to PLOS's standards.

At this time, please upload the minimal data set necessary to replicate your study's findings to a stable, public repository (such as figshare or Dryad) and provide us with the relevant URLs, DOIs, or accession numbers that may be used to access these data. For a list of recommended repositories and additional information on PLOS standards for data deposition, please see <https://journals.plos.org/plosone/s/recommended-repositories>.

**Response:** We appreciate this important point. The original audio dataset used in this study is available to approved researchers through the Korean AI Hub Safe Zone (https://safezone.aihub.or.kr/). Due to participant privacy regulations, unrestricted public distribution of the original audio recordings is not possible. However, the data is openly accessible to any global researcher upon application for scholarly purposes. To reproduce our findings, interested scientists can formally access the precise identical dataset by registering at the AI Hub Safe Zone (<https://safezone.aihub.or.kr/> ) and electronically agreeing to the ethical terms of use.

To comply with PLOS's data sharing standards, we have deposited the core source code necessary for reproducing our study's findings on figshare as a private item. A private sharing link has been provided to reviewers (<https://figshare.com/s/3d395292a035369d4bac>), The deposited code includes:

- Complete implementations of all 11 model architectures (1D CNN, AST, Wav2Vec 2.0, and 6 ImageNet-pretrained CNNs)

- The training pipeline (AdamW optimizer, cosine annealing warm restarts, mixed-precision training, early stopping)

- The audio preprocessing pipeline (Bessel bandpass filtering, spectrogram/mel-spectrogram/MFCC extraction)

- Evaluation utilities (classification reports, confusion matrices, k-fold cross-validation aggregation)

7. PLOS requires an ORCID iD for the corresponding author in Editorial Manager on papers submitted after December 6th, 2016. Please ensure that you have an ORCID iD and that it is validated in Editorial Manager. To do this, go to ‘Update my Information’ (in the upper left-hand corner of the main menu), and click on the Fetch/Validate link next to the ORCID field. This will take you to the ORCID site and allow you to create a new iD or authenticate a pre-existing iD in Editorial Manager.

**Response:** As your recommendation, My ORCID (0000-0002-2742-1759) have been authenticated and updated.

8. Your ethics statement should only appear in the Methods section of your manuscript. If your ethics statement is written in any section besides the Methods, please move it to the Methods section and delete it from any other section. Please ensure that your ethics statement is included in your manuscript, as the ethics statement entered into the online submission form will not be published alongside your manuscript.

**Response:** We have relocated the ethics statement straight to the 'Methods' section and deleted it from the 'Declarations' section at the end of the manuscript.

9. If the reviewer comments include a recommendation to cite specific previously published works, please review and evaluate these publications to determine whether they are relevant and should be cited. There is no requirement to cite these works unless the editor has indicated otherwise. 

**Additional Editor Comments:**

Reviewers' comments:

Reviewer's Responses to Questions

**Comments to the Author**

1. Is the manuscript technically sound, and do the data support the conclusions?

The manuscript must describe a technically sound piece of scientific research with data that supports the conclusions. Experiments must have been conducted rigorously, with appropriate controls, replication, and sample sizes. The conclusions must be drawn appropriately based on the data presented.

Reviewer #1: Yes

Reviewer #2: Yes

2. Has the statistical analysis been performed appropriately and rigorously?

Reviewer #1: No

Reviewer #2: Yes

3. Have the authors made all data underlying the findings in their manuscript fully available?

The [PLOS Data policy](http://track.editorialmanager.com/CL0/http:%2F%2Fwww.plosone.org%2Fstatic%2Fpolicies.action%23sharing/1/010f019c55fdd233-6e527ef8-6f32-4ad9-8ab2-f78796f516b1-000000/7JbqEprnL4s3154AHRO-ufZKhGsrifZ7IhwAuhLWPqc=250) requires authors to make all data underlying the findings described in their manuscript fully available without restriction, with rare exception (please refer to the Data Availability Statement in the manuscript PDF file). The data should be provided as part of the manuscript or its supporting information, or deposited to a public repository. For example, in addition to summary statistics, the data points behind means, medians and variance measures should be available. If there are restrictions on publicly sharing data—e.g. participant privacy or use of data from a third party—those must be specified.

Reviewer #1: Yes

Reviewer #2: Yes

4. Is the manuscript presented in an intelligible fashion and written in standard English?

PLOS ONE does not copyedit accepted manuscripts, so the language in submitted articles must be clear, correct, and unambiguous. Any typographical or grammatical errors should be corrected at revision, so please note any specific errors here.

Reviewer #1: No

Reviewer #2: Yes

5. Review Comments to the Author

Please use the space provided to explain your answers to the questions above. You may also include additional comments for the author, including concerns about dual publication, research ethics, or publication ethics. (Please upload your review as an attachment if it exceeds 20,000 characters)

**Reviewer #1:** This manuscript explores an important and emerging field of speech-based detection of cognitive impairment using deep learning models. The comparative approach across multiple architectures (1D CNN, AST, Wav2Vec 2.0, etc.) is valuable and well-aligned with the journal’s scope. However, the study requires major revisions to improve methodological transparency, statistical rigor, interpretability, and presentation clarity before it can be considered for publication.

1. Clarify dataset composition, total participants, and inclusion of all 11 speech tasks.

**Response:** We thank the reviewer for identifying this ambiguity. We have comprehensively revised the 'Methods - Dataset' section. We now explicitly state the absolute number of total participants analyzed, and we clarified that the audio recordings spanning all 11 heterogeneous speech tasks were aggregated and utilized collectively per participant to generate sufficient temporal data for deep learning algorithms.

“**Dataset**

The dataset used in this study was acquired from the "Cognitive Impairment Diagnosis Voice/Conversation" offline data on 07/09/2022, which can be accessed at the AI Hub Safe Zone (https://safezone.aihub.or.kr/). This dataset contains voice data from individuals with dementia and control groups, collected through the SpeechTaskSet application. This clinical app is structured to guide participants through 11 heterogeneous speech tasks, including sentence repetition (three tasks), image description (two tasks), language fluency (two tasks), calculation (one task), and storytelling (three tasks), producing an audio file of up to 1 min per task. Notably, the app captures only the voices of the participants, excluding any input from the instructors. To generate sufficient temporal data for deep learning algorithms, the audio recordings spanning all 11 speech tasks were aggregated and utilized collectively per participant.” … “The AI Hub dataset included 3,483 voice recordings from 320 female participants and 1,481 from 136 male participants. Owing to the lower volume of male recordings, this study prioritized the female voice dataset for model training to prevent extreme class imbalance. A total of 320 female participants were analyzed, including 105 with AD, 92 with MCI, and 123 cognitively normal controls (NC) (Table 1). The recordings from the AD group corresponded to an average age of 72 years (57-90 y). The corresponding value for the MCI group was 75 years (53-93 y), and for the NC group, it was 69 years (54-88 y).”

2. Address data imbalance (e.g., female-majority dataset) and its effect on generalization.

**Response:** This is a vital methodological point. We initially prioritized the female cohort solely to prevent extreme class imbalance, given the severe lack of available male recordings across target cognitive stages in the AI Hub repository. We entirely concur that relying on a female-majority dataset restricts the robust generalization of our trained weights to male demographics, because gender-specific intrinsic vocal characteristics like fundamental frequency and formants impact feature mappings. We have incorporated a critical paragraph inside the Discussion detailing this severe limitation, explicitly proposing subsequent studies incorporating gender-balanced demographic parity. We inserted the following paragraph into the Discussion section just before the "Conclusions" heading:

" Several methodological limitations of this study must be addressed to appropriately contextualize our findings. First, regarding dataset size, while our cross-validation strategy rigorously ensured internal robustness, the overall participant count (N=320) remains relatively limited for training complex transformer architectures like Wav2Vec 2.0. A larger cohort is necessary to fully exploit model capacity and prevent subtle overfitting. Second, we acknowledge a critical gender bias within our sample. We initially prioritized the female cohort solely to prevent extreme class imbalance, given the limited availability of male recordings across target cognitive stages in the AI Hub repository. We acknowledge that relying on a female-only dataset limits the generalizability of our findings to male populations, because gender-specific vocal characteristics such as fundamental frequency and formants significantly affect acoustic feature representations. Consequently, future studies should incorporate gender-balanced cohorts to ensure comprehensive and equitable diagnostic applicability. Finally, the models were trained exclusively on native Korean speakers. Because phonetics, articulation rates, and pausing intervals intrinsically vary across languages, the current findings may not generalize directly to other linguistic populations. Future multinational-scale validations are required.”

3. Describe preprocessing steps such as noise reduction, silence trimming, and normalization.

**Response:** We thank the reviewer for this important point. We have added a comprehensive description of the audio preprocessing pipeline in the Methods section. Specifically, the following preprocessing steps were applied:

**“Audio Preprocessing**

(1) Audio loading and resampling: All audio files were loaded as 32-bit floating-point signals using the soundfile library and resampled to 16 kHz using librosa, in accordance with the Nyquist–Shannon sampling theorem. Multi-channel recordings were converted to mono by channel averaging.

(2) Bandpass filtering: To attenuate environmental noise and frequency components outside the human speech frequency range, a fifth-order Bessel bandpass filter (300–4000 Hz) was applied to all audio signals. This filtering procedure served as the primary signal cleaning mechanism, replacing a dedicated noise reduction algorithm by constraining the frequency band to the speech-relevant range.

(3) Duration filtering: Recordings shorter than 3 seconds were excluded based on empirical observation that such brief recordings are disproportionately affected by background noise. This criterion ensures a minimum threshold of meaningful speech content for analysis.

(4) Feature-level normalization: During spectrogram generation, the magnitude spectrum was converted to a power spectrum and subsequently transformed to the decibel (dB) scale. Reference normalization against the maximum power was applied (amin=1e-16), the dynamic range was clipped to 80 dB, and the resulting values were linearly normalized to the [0, 1] range.

No explicit silence trimming algorithm was applied. This design choice was intentional, as pause patterns and their frequency during speech are considered potential diagnostic indicators of cognitive impairment in this study. Pausing behavior—including the duration, frequency, and distribution of silent intervals—may reflect underlying cognitive decline and was therefore preserved as an informative feature in the model input.”

4. Specify details of data splits and confirm participant-level separation in cross-validation.

**Response:** We appreciate the reviewer's request for clarification. We have added detailed descriptions of the data split procedure and participant-level separation in the Model Evaluation section.

The cross-validation was designed with strict participant-level separation. Specifically:

- All recordings from each participant (identified by unique provider ID) were assigned to a single fold, preventing any data leakage where the same participant's recordings could appear in both training and validation sets simultaneously.

- The fold assignment was stratified by cognitive status label, ensuring that each fold maintained a similar proportion of NC, MCI, and AD participants as the overall dataset.

- To mitigate class imbalance, an under-sampling strategy was employed. The number of participants per class was balanced to match the minority class, with gradual per-participant data point adjustment to achieve balanced class representation within each fold.

- Cross-validation was performed independently for each binary classification task (NC vs. MCI and NC vs. AD).

‘Specifically, the fold assignment was stratified by cognitive status label to ensure that each fold maintained a similar proportion of participants from each diagnostic group. An under-sampling strategy was employed to balance class representation: the number of participants per class within each fold was adjusted to match the minority class, with gradual per-participant data point adjustment to achieve equitable class distributions. This participant-level separation guaranteed that all recordings from a given individual appeared exclusively in either the training or validation set within any given fold, thereby eliminating data leakage and ensuring that the model generalized based on disorder-related acoustic features rather than memorizing individual speaker characteristics. Each binary classification task (NC vs. MCI and NC vs. AD) was evaluated independently through this cross-validation framework. To assess the statistical significance of performance differences between models, pairwise proportion z-tests were conducted comparing each model's classification accuracy against that of Wav2Vec 2.0 (the best-performing model). Since each participant was assigned to exactly one test fold in the five-fold cross-validation, the aggregated accuracy across all folds represents the overall proportion of correctly classified participants. Wilson score intervals were computed to obtain 95% confidence intervals (CIs) for each model's accuracy. To control the family-wise error rate across multiple pairwise comparisons, Holm-Bonferroni correction was applied to the resulting p-values. Cohen's h effect size was computed for each comparison to quantify the magnitude of performance differences. A significance threshold of α = 0.05 was adopted.’

5. Provide hyperparameter tuning details (learning rate, epochs, optimizer, batch size).

**Response:** Following the reviewer's request, we have provided a comprehensive description of the training configurations for all models in the Methods section. The key hyperparameters are as follows:

- Optimizer: AdamW (Adam variant with decoupled weight decay regularization)

- Loss function: Cross-entropy loss (for binary classification)

- Base learning rate: 5 × 10⁻⁵

- Learning rate scheduler: Cosine Annealing Warm Restarts (T₀ = 20,000 steps, T_mult = 2, η_min = 1 × 10⁻⁵)

- Batch size: 1 per GPU step with gradient accumulation over 16 steps (effective batch size of 16)

- Mixed-precision training: FP16 operations using PyTorch's autocast and GradScaler

- Gradient clipping: L2 norm with max_norm = 2.0

- Early stopping: Based on validation loss with a patience of 10 consecutive validation checks without improvement

All models shared these core training hyperparameters to ensure a fair and consistent comparison across architectures. The only architecture-specific differences were inherent to the model structures themselves (e.g., Wav2Vec 2.0's frozen feature extractor and feature projection layers).

‘**Training Configuration**

All deep learning models were trained with a unified set of core hyperparameters to ensure fair comparison. The AdamW optimizer (Adam with decoupled weight decay regularization) was employed with a base learning rate of 5 × 10⁻⁵. A cosine annealing warm restarts learning rate scheduler was applied (T₀ = 20,000 steps, T_mult = 2, η_min = 1 × 10⁻⁵) to facilitate stable convergence. The batch size was set to 1 per GPU step with gradient accumulation over 16 steps, yielding an effective batch size of 16. Mixed-precision training was utilized via PyTorch's automatic mixed precision (autocast and GradScaler) to reduce memory consumption and accelerate computation. Gradient clipping with an L2 norm constraint of 2.0 was applied to prevent gradient explosion. The loss function used was binary cross-entropy (BCE) loss. An early stopping strategy based on validation loss was implemented with a patience of 10 epochs without improvement. All experiments were conducted on a system equipped with an NVIDIA Geforce RTX 3090 GPU (24 GB memory) and a CPU with 32 GB of RAM. The GPU memory requirements varied by model architecture: Wav2Vec 2.0 required approximately 23 GB, the AST (Transformer) required approximately 20 GB, and the 1D CNN and image-based pre-trained models (AlexNet, VGG-19, ResNet, DenseNet, Inception, SqueezeNet) each required approximately 10 GB of GPU memory.’

6. Add statistical tests to verify significance of model performance differences.

**Response:** We thoroughly agree that statistical validation enhances scientific rigor. Following the reviewer's feedback, we performed the following comprehensive statistical analyses:

(1) Wilson score 95% confidence intervals (CIs): Wilson score intervals were computed for each model's classification accuracy. This method provides accurate CIs for binomial proportions and offers superior coverage compared to Wald intervals when sample sizes are limited.

(2) Pairwise proportion z-tests: Approximate proportion z-tests were conducted comparing Wav2Vec 2.0's accuracy (best-performing model) against each competing model.

(3) Holm-Bonferroni multiple comparison correction: To control the family-wise error rate across 10 pairwise comparisons, Holm-Bonferroni correction was applied to all p-values.

(4) Cohen's h effect sizes: Cohen's h was computed for each pairwise comparison to quantify the magnitude of practical differences.

Key findings:

- In the NC vs. AD task, Wav2Vec 2.0 (accuracy 0.83, 95% CI [0.776, 0.873]) significantly outperformed 7 of 10 competing models (corrected p < 0.05): 1D CNN Spectrogram (p = 0.014), Mel-Spectrogram (p < 0.001), MFCC (p < 0.001), AST (p < 0.001), AlexNet (p = 0.025), Inception (p < 0.001), and ResNet (p = 0.040).

- VGG-19 (p = 0.058), DenseNet (p = 0.356), and SqueezeNet (p = 0.409) did not differ significantly.

- Cohen's h effect sizes ranged from 0.08 (SqueezeNet) to 0.69 (AST), indicating small to medium practical differences.

These inferential statistical results have been incorporated into the revised Results text and Table 2.

In the methods:

‘To assess the statistical significance of performance differences between models, pairwise proportion z-tests were conducted comparing each model's classification accuracy against that of Wav2Vec 2.0 (the best-performing model). Since each participant was assigned to exactly one test fold in the five-fold cross-validation, the aggregated accuracy across all folds represents the overall proportion of correctly classified participants. Wilson score intervals were computed to obtain 95% confidence intervals (CIs) for each model's accuracy. To control the family-wise error rate across multiple pairwise comparisons, Holm-Bonferroni correction was applied to the resulting p-values. Cohen's h effect size was computed for each comparison to quantify the magnitude of performance differences. A significance threshold of α = 0.05 was adopted.’

In the results session:

‘Pairwise proportion z-tests with Holm-Bonferroni correction confirmed that Wav2Vec 2.0 significantly outperformed the majority of competing models. In the NC vs. AD task, 7 of 10 pairwise comparisons reached statistical significance (corrected p < 0.05): 1D CNN Spectrogram (p = 0.014), Mel-Spectrogram (p < 0.001), MFCC (p < 0.001), AST (p < 0.001), AlexNet (p = 0.025), Inception (p < 0.001), and ResNet (p = 0.040). Three models did not differ significantly from Wav2Vec 2.0: VGG-19 (p = 0.058), DenseNet (p = 0.356), and SqueezeNet (p = 0.409). Cohen's h effect sizes ranged from 0.08 (SqueezeNet) to 0.69 (AST), indicating small to medium practical differences. Wilson score 95% confidence intervals for all model accuracies are reported in Table 2.’

7. Present confusion matrices or performance plots for key models.

**Response:** We strongly agree that qualitative visual representations promote interpretability. We have generated confusion matrices for the top-performing Wav2Vec 2.0 model and the baseline 1D CNN (Spectrogram) model, covering both the NC vs. MCI and NC vs. AD binary classification tasks. These confusion matrices, aggregated across all folds of the 5-fold cross-validation, have been added as Figure 4.

Specific results:

- (A) Wav2Vec 2.0, NC vs. MCI: 69 of 92 MCI correctly classified (sensitivity 0.75), 90 of 123 NC correctly classified (specificity 0.73)

- (B) Wav2Vec 2.0, NC vs. AD: 87 of 105 AD correctly classified (sensitivity 0.83), 102 of 123 NC correctly classified (specificity 0.83)

- (C) 1D CNN, NC vs. MCI: 60 of 92 MCI correctly classified (sensitivity 0.65), 80 of 123 NC correctly classified (specificity 0.65)

- (D) 1D CNN, NC vs. AD: 75 of 105 AD correctly classified (sensitivity 0.71), 87 of 123 NC correctly classified (specificity 0.71)

The confusion matrices for Wav2Vec 2.0 demonstrate a well-balanced sensitivity-specificity profile, particularly pronounced in the AD classification task, reflecting the more distinct speech changes associated with AD.

‘

Fig 4. Confusion matrices for the Wav2Vec 2.0 and 1D CNN (Spectrogram) models across binary classification tasks. (A) Wav2Vec 2.0 for NC vs. MCI. (B) Wav2Vec 2.0 for NC vs. AD. (C) 1D CNN (Spectrogram) for NC vs. MCI. (D) 1D CNN (Spectrogram) for NC vs. AD. Values represent aggregated predictions across all five cross-validation folds. NC, Normal Cognition; MCI, Mild Cognitive Impairment; AD, Alzheimer's Disease.

Figure 4 presents the confusion matrices for the Wav2Vec 2.0 and 1D CNN (Spectrogram) models based on aggregated predictions across all five cross-validation folds. For the Wav2Vec 2.0 model, the NC vs. MCI classification (Fig 4A) correctly identified 69 out of 92 MCI cases and 90 out of 123 NC cases, yielding a sensitivity of 0.75 and a specificity of 0.73. In the NC vs. AD task (Fig 4B), the model achieved higher performance with a sensitivity of 0.83 (87/105 AD) and a specificity of 0.83 (102/123 NC).

In contrast, the 1D CNN (Spectrogram) model showed lower classification accuracy. In the NC vs. MCI task (Fig 4C), it correctly identified 60 MCI and 80 NC cases (sensitivity 0.65, specificity 0.65). Similarly, for the NC vs. AD classification (Fig 4D), the sensitivity and specificity were both 0.71, with 75 of 105 AD and 87 of 123 NC cases correctly predicted.’

8. Compare with simpler baseline models (e.g., SVM or logistic regression with MFCC).

**Response:** We appreciate the reviewer's emphasis on the importance of a traditional machine learning baseline.

Our study already includes the 1D CNN (MFCC) model, which serves as a bridge between traditional handcrafted-feature approaches and deep learning. MFCC features are the most widely used standard features in SVM-based speech classification, and our 1D CNN (MFCC) model utilizes identical 13-dimensional MFCC features as input. It achieved an accuracy of 0.66 in both NC vs. MCI and NC vs. AD tasks, significantly lower than Wav2Vec 2.0 (0.74 and 0.83, respectively), with statistically significant differences confirmed (NC vs. AD: p < 0.001 after Holm-Bonferroni correction).

These results demonstrate that handcrafted feature engineering alone is insufficient for capturing the complex acoustic patterns associated with cognitive impairment, supporting the intrinsic diagnostic value of Wav2Vec 2.0's self-supervised representation learning. This analysis has been added to the Discussion section.

‘The notably lower accuracy of models relying on simplified feature representations (e.g., 1D CNN with MFCC achieving 0.66 in both tasks) compared to Wav2Vec 2.0 underscores that manual feature engineering alone is insufficient for capturing the complex acoustic patterns associated with cognitive impairment. This observation supports the intrinsic diagnostic value of automated representation learning, particularly the self-supervised approach employed by Wav2Vec 2.0, which can discover latent speech features without explicit feature design.’

9. Expand limitations regarding dataset size, gender bias, and language specificity.

**Response:** I have drafted the expanded limitations covering dataset size, gender bias, and language specificity. Here is the exact text paragraph I generated to replace the current Limitation section:

“Several methodological limitations of this study must be addressed to appropriately contextualize our findings. First, regarding dataset size, while our cross-validation strategy rigorously validated internal robustness, the overall participant count (N=320) remains relatively constrained for deeply training complex transformer architectures like Wav2Vec 2.0. A larger cohort is necessary to fully saturate model capacity and prevent subtle overfitting. Second, we acknowledge a critical gender bias within our sample. We initially prioritized the female cohort solely to prevent extreme class imbalance, given the severe lack of available male recordings across target cognitive stages in the AI Hub repository. We entirely concur that relying on a female-majority dataset restricts the robust generalization of our trained weights to male demographics, because gender-specific intrinsic vocal characteristics like fundamental frequency and formants drastically impact feature mappings. Consequently, subsequent studies must incorporate gender-balanced demographic parity to ensure comprehensive and equitable diagnostic applicability. Finally, the models were trained exclusively on native Korean speakers. Because phonetics, articulation rates, and pausing intervals intrinsically vary across languages, the current diagnostic thresholds lack direct language specificity generalization to other linguistic populations. Future multinational scale validations are required."

10. Add DOI: 10.1109/OJCS.2025.3580570, this study’s use of 1D-to-2D transformations (CWT, STFT) and CNN-based modelling parallels the present work’s spectrogram approach, reinforcing its methodological validity and connecting speech-based cognitive analysis to broader applications in noninvasively biomedical signal processing.

**Response:** We carefully reviewed the indicated literature. We agree that its methodology closely mirrors our localized spectrogram architecture and elegantly contextualizes speech-based predictive diagnostics within the landscape of biomedical signal processing. We added the following text to the end of the paragraph introducing 1D CNNs and spectrograms:

"This methodological transition from 1D sequential audio signals to structured 2D visual representations (spectrograms) for complex pattern recognition closely mirrors advanced signal processing paradigms emerging in other biomedical domains, such as utilizing 1D-to-2D transformations coupled with CNNs for radio frequency-based emotion identification [18]."

"18. AbuAli N, Jadoon I, Ullah F, Hayajneh M, Alkobaisi S. Radio Frequency SensingBased Human Emotion Identification by Leveraging 2D Transformation Techniques and Deep Learning Models. IEEE Open Journal of the Computer Society. 2025;6:1178-1189."

12. Add model complexity metrics (parameters, training time, memory).

**Response:** We appreciate the reviewer's suggestion. We have added the computing environment information necessary for experimental reproducibility to the Training Configuration subsection within Methods. Specifically, all experiments were conducted on a system with 24 GB GPU memory and 32 GB CPU RAM. The GPU memory requirements varied by model: Wav2Vec 2.0 required approximately 23 GB, the AST (Transformer) approximately 20 GB, and the 1D CNN and image-based pre-trained models (AlexNet, VGG-19, ResNet, DenseNet, Inception, SqueezeNet) approximately 10 GB each. We deemed the Training Configuration section a more methodologically appropriate location for this reproducibility information than the Discussion.

‘**Training Configuration**

All deep learning models were trained with a unified set of core hyperparameters to ensure fair comparison. The AdamW optimizer (Adam with decoupled weight decay regularization) was employed with a base learning rate of 5 × 10⁻⁵. A cosine annealing warm restarts learning rate scheduler was applied (T₀ = 20,000 steps, T_mult = 2, η_min = 1 × 10⁻⁵) to facilitate stable convergence. The batch size was set to 1 per GPU step with gradient accumulation over 16 steps, yielding an effective batch size of 16. Mixed-precision training was utilized via PyTorch's automatic mixed precision (autocast and GradScaler) to reduce memory consumption and accelerate computation. Gradient clipping with an L2 norm constraint of 2.0 was applied to prevent gradient explosion. The loss function used was binary cross-entropy (BCE) loss. An early stopping strategy based on validation loss was implemented with a patience of 10 epochs without improvement. All experiments were conducted on a system equipped with an NVIDIA Geforce RTX 3090 GPU (24 GB memory) and a CPU with 32 GB of RAM. The GPU memory requirements varied by model architecture: Wav2Vec 2.0 required approximately 23 GB, the AST (Transformer) required approximately 20 GB, and the 1D CNN and image-based pre-trained models (AlexNet, VGG-19, ResNet, DenseNet, Inception, SqueezeNet) each required approximately 10 GB of GPU memory.’

13. Strengthen abstract, improve figure captions, and edit text.

**Response:** We have enriched the Abstract summarizing absolute dataset proportions. Figure captions were structurally fortified maximizing clarity.

Abstract (Methods): “The study analyzed voice recordings from 320 female participants (105 with Alzheimer's disease, 92 with mild cognitive impairment, and 123 cognitively normal controls).”

Figure Captions:

Fig 1. Architecture of the 1D CNN (1-Dimensional Convolutional Neural Network) model. The diagram illustrates the sequential transformation of raw audio features through feature extraction layers down to the final classification logic, highlighting its capability to process sequential time-series acoustic data.

Fig 2. Architecture of the AST (Audio Spectrogram Transformer) model. This schematic details how 2D audio spectrograms are divided into localized patches and processed through transformer encoder blocks utilizing self-attention mechanisms to learn global acoustic context.

Fig 3. Architecture of the Wav2Vec 2.0 model. The figure demonstrates the self-supervised learning pipeline, showcasing the initial encoding of raw speech waveforms via a CNN block followed by deep contextualized representation learning within a robust transformer network.

Fig 4. Confusion matrices for the Wav2Vec 2.0 and 1D CNN (Spectrogram) models across binary classification tasks. (A) Wav2Vec 2.0 for NC vs. MCI. (B) Wav2Vec 2.0 for NC vs. AD. (C) 1D CNN (Spectrogram) for NC vs. MCI. (D) 1D CNN (Spectrogram) for NC vs. AD. Values represent aggregated predictions across all five cross-validation folds. NC, Normal Cognition; MCI, Mild Cognitive Impairment; AD, Alzheimer's Disease.

**Reviewer #2:** This study addresses the critical need for no-invasive diagnostic tools for cognitive decline, by evaluating multiple deep learning architectures applied to dataset from “Cognitive Impairment Diagnosis Voice/Conversation”. Using this dataset the study compares the efficacy of 1D CNN, AST and Wav2Vec2.0 in classifying binary tasks: normal cognition (NC) vs. mild cognitive impairment (MCI) and NC vs. Alzheimer’s disease (AD). The paper concludes that Wav2Vec2.0 outperforms other architectures, particularly in the NC vs. AD classification task.

The primary strength of this work lies in systematic, side by side evaluation of disparate modeling approaches (eg. VGG-19, ResNet, transformer-based models) to audio models providing a clear hierarchy of effectiveness. In particular, the results reveal that self-supervised speech representation model such as Wav2Vec2.0 is better suited for cognitive changes in speech.

I had a few comments that I believe would improve the paper and its applicability.

1.This study prioritizes female voices due to lower volume of male recordings. Although this choice is understandable, it raises concern about generalizability of the results. It would have been interesting to see how sex-specific vocal characteristics might influence model performance and whether conclusions are expected to hold for equally mixed/male-dominated cohorts.

**Response:** We deeply appreciate the Reviewer highlighting this profound constraint. As detailed extensively in our response to Reviewer #1, focusing purely on female profiles drastically impairs direct inference to mixed demographic populations governed by inherently different pathological vocal profiles. We have incorporated a critical paragraph illuminating this phenomenon, stressing that sex-specific vocal physiology necessitates future model calibration focusing equally on male-dominated biological expressions inside the Discussion. We inserted the following paragraph into the Discussion section just before the "Conclusions" heading:

" Several methodological limitations of this study must be addressed to appropriately contextualize our findings. First, regarding dataset size, while our cross-validation strategy rigorously ensured internal robustness, the overall participant count (N=320) remains relatively limited for training complex transformer architectures like Wav2Vec 2.0. A larger cohort is necessary to fully exploit model capacity and prevent subtle overfitting. Second, we acknowledge a critical gender bias within our sample. We initially prioritized the female cohort solely to prevent extreme class imbalance, given the limited availability of male recordings across target cognitive stages in the AI Hub repository. We acknowledge that relying on a female-only dataset limits the generalizability of our findings to male populations, because gender-specific vocal characteristics such as fundamental frequency and formants significantly affect acoustic feature representations. Consequently, future studies should incorporate gender-balanced cohorts to ensure comprehensive and equitable diagnostic applicability. Finally, the models were trained exclusively on native Korean speakers. Because phonetics, articulation rates, and pausing intervals intrinsically vary across languages, the current findings may not generalize directly to other linguistic populations. Future multinational-scale validations are required.”

2. If I could make out correctly, the results aggregate the performance across the 11 different tasks (eg. Sentence repetition, language fluency). In the limiting case what would be the effect of analyzing these individually? Some tasks (eg. storytelling) are biologically more relevant for cognitive decline than other (Image description). I was wondering which specific tasks account for higher Wav2Vec2.0. This in turn could streamline future data collections.

**Response:** This is an exceptionally brilliant insight predicting our subsequent research trajectory. Our extant paradigm deliberately amalgamated the 11 tasks simply to aggregate continuous longitudinal acoustic features. However, we profoundly agree that particular verbal prompts (e.g., intricate storytelling versus simple repetition) invoke disparate cognitive faculties, inevitably manifesting distinct biological markers dictating neurodegeneration. In the future study, we will explicitly formulate this hypothesis, stressing that functionally isolating discrete cognitive tasks to identify optimal conversational biomarkers represents the paramount frontier to definitively streamline diagnostic data collection. The following text was inserted into the Discussion section, right after the limitations paragraph and exactly before the "Conclusions" heading

" A further direction for future research involves the analysis of the 11 individual speech tasks. Our current approach combined these tasks to aggregate continuous acoustic features. However, different verbal prompts (e.g., complex storytelling versus simple repetition) engage distinct cognitive faculties, potentially yielding different biomarkers associated with neurodegeneration. In a future study, we will test this hypothesis, emphasizing that isolating individual cognitive tasks to identify the most informative speech biomarkers represents an important direction for streamlining diagnostic data collection."

3.Finally, the model performance is reported using across five-fold cross-validation. Given relatively modest performance differences between some models, reporting statistical significance testing/confidence interval would help clarify the robustness.

**Response:** Thank you for urging us to ensure inferential clarity. We have computed rigorous Wilson score 95% confidence intervals (CIs) for classification accuracy aggregated across the 5-fold cross-validation, accompanied by explicit statistical significance measures using pairwise proportion z-tests with Holm-Bonferroni correction. Cohen's h effect sizes were also computed to quantify the magnitude of practical differences.

Key findings: Wav2Vec 2.0 (accuracy 0.83, 95% CI [0.776, 0.873]) significantly outperformed 7 of 10 competing models in the NC vs. AD task (corrected p < 0.05). Wilson score CIs and statistical test results have been incorporated into the revised Results text and Table 2.

Overall, this manuscript presents a well-executed study using speech dataset and deep learning techniques. The comparative analysis is valuable contribution to the field of digital biomarker. I believe addressing above points would strengthen the paper.

6. PLOS authors have the option to publish the peer review history of their article ([what does this mean?](https://track.editorialmanager.com/CL0/https:%2F%2Fjournals.plos.org%2Fplosone%2Fs%2Feditorial-and-peer-review-process%23loc-peer-review-history/1/010f019c55fdd233-6e527ef8-6f32-4ad9-8ab2-f78796f516b1-000000/hELTubBARJRya7UG5j-2koM9Uu0G1HD_wawm9BozxP8=250)). If published, this will include your full peer review and any attached files.

If you choose “no”, your identity will remain anonymous but your review may still be made public.

**Do you want your identity to be public for this peer review?** For information about this choice, including consent withdrawal, please see our [Privacy Policy](https://track.editorialmanager.com/CL0/https:%2F%2Fwww.plos.org%2Fprivacy-policy/1/010f019c55fdd233-6e527ef8-6f32-4ad9-8ab2-f78796f516b1-000000/xXTSOPu4uQrZ7KZBubiubI5v9xWK6WwH6Fa4fsn4Y6c=250).

Reviewer #1: No

Reviewer #2: No
